# Ablative Five-Fraction CT Versus MR-Guided Stereotactic Body Radiation Therapy for Pancreatic Cancer: In Silico Evaluation of Interfraction Anatomic Changes as a Rationale for Online Adaptive Replanning

**DOI:** 10.3390/cancers17132061

**Published:** 2025-06-20

**Authors:** Adeel Kaiser, Nicole Luther, Kathryn E. Mittauer, Amna Gul, Robert A. Herrera, Mukesh K. Roy, Ashley Fellows, Amy Rzepczynski, Will Deere, Matthew D. Hall, Rupesh Kotecha, Nema Bassiri-Gharb, Alonso N. Gutierrez, Michael D. Chuong

**Affiliations:** 1 Department of Radiation Oncology, Miami Cancer Institute, Miami, FL 33176, USA; nicolemca@baptisthealth.net (N.L.); kathrynm@baptisthealth.net (K.E.M.); amna.gul@baptisthealth.net (A.G.); robertoherr@baptisthealth.net (R.A.H.); mukesh.roy@baptisthealth.net (M.K.R.); ashley.fellows@baptisthealth.net (A.F.); amyrz@baptisthealth.net (A.R.); willde@baptisthealth.net (W.D.); matthewha@baptisthealth.net (M.D.H.); rupeshk@baptisthealth.net (R.K.); bassirin@uthscsa.edu (N.B.-G.); alonsog@baptisthealth.net (A.N.G.); michaelchu@baptisthealth.net (M.D.C.); 2 Herbert Wertheim College of Medicine, Florida International University, Miami, FL 33199, USA

**Keywords:** pancreatic cancer, pancreatic adenocarcinoma, stereotactic radiation, MR-guided radiotherapy, adaptive radiotherapy, ablative radiotherapy, SBRT, SMART

## Abstract

**Background/Objectives:** Non-ablative stereotactic body radiation therapy (SBRT) is commonly employed for locally advanced pancreatic cancer (LAPC) using computed tomography-guided radiotherapy (CTgRT) without online adaptive radiation therapy (oART). The safe delivery of ablative SBRT has been demonstrated using stereotactic magnetic resonance-guided online adaptive radiation therapy (SMART). We performed an in silico comparison of non-adapted CTgRT versus SMART to better understand the potential benefit of oART for ablative pancreatic SBRT. **Methods:** We retrospectively evaluated original and daily adapted SMART plans that were previously delivered for 20 consecutive LAPC cases (120 total plans across all patients) treated on a 0.35 T MR-linac prescribed to 50 Gy (gross disease) and 33 Gy (elective sites) simultaneously in five fractions. Six comparative CTgRT plans for each patient (one original, five daily treatment) were retrospectively generated with the same prescribed dose and planning parameters as the SMART plans assuming no oART availability. The impact of daily anatomic changes on CTgRT and SMART plans without oART was evaluated across each treatment day MRI scan acquired for SMART. **Results:** Ninety percent of cases involved the pancreatic head. No statistically significant differences were seen between CTgRT and SMART with respect to target coverage. Nearly all (96%) fractions planned on either CT or MRI platforms exceeded at least one GI organ at risk (OAR) constraint without oART. Significant differences favoring SMART over non-adaptive CTgRT were observed for the duodenum V35 Gy ≤ 0.5 cc (34.2 vs. 41.9 Gy, *p* = 0.0035) and duodenum V40 Gy ≤ 0.03 cc (37 vs. 52.5 Gy, *p* = 0.0006) constraints. Stomach V40 Gy trended towards significance favoring SMART (37 vs. 40.3 Gy, *p* = 0.057) while no significant differences were seen. **Conclusions:** This is the first study that quantifies the frequency and extent of GI OAR constraint violations that would occur during ablative five-fraction SBRT using SMART vs. CTgRT. GI OAR constraint violations are expected for most fractions without oART whereas all constraints can be achieved with oART. As such, these data suggest that oART should be required for ablative five-fraction pancreatic SBRT.

## Simple Summary

Prospective data has now demonstrated superior outcomes for locally advanced pancreatic cancer patients treated with ablative rather than non-ablative stereotactic body radiation therapy (SBRT). MRI guided, online adaptive radiotherapy (oART) strategies have been employed to limit radiation doses and toxicity to organs at risk (OAR) during ablative SBRT. Given the paucity of cancer centers with oART capability, we compared non-adapted, CT-guided radiation plans with MR-guided plans using oART across all treatment fractions during ablative SBRT. Plan comparison was completed using pre-treatment MRI imaging and demonstrated significantly higher doses to organs at risk in the absence of oART. These results suggest the need to limit ablative SBRT to centers with oART capability.

## 1. Introduction

In 2025, approximately 67,000 new diagnoses and 52,000 deaths are estimated in the United States from pancreatic ductal adenocarcinomas (PDACs) [1]. PDAC remains the fourth leading cause of cancer-related death in men and has now overtaken colorectal cancer as the third most common cause of cancer-related death in women. Roughly 35% of patients present with locally advanced pancreatic cancer (LAPC), not amenable to surgery but without radiographic evidence of metastatic disease [2]. Despite the risk of metastatic spread in this patient population, up to approximately one-third of patients may die from complications related to local disease progression. This highlights the importance of achieving durable local control (LC), particularly in the subset of patients who harbor genetic predispositions to higher rates of LC without distant metastases, such as in SMAD4 expressing tumors [2,3,4].

Radiation therapy (RT) has been prospectively demonstrated to improve LC in LAPC [5]. However, conventional CT-guided radiation therapy (CTgRT) is typically non-ablative with biologically effective doses (BED_10_) below at least 70 Gy to minimize toxicity due to a high frequency of interfraction anatomic changes especially related to gastrointestinal (GI) luminal organs at risk (OARs), the lack of routine intrafraction imaging, and suboptimal soft tissue contrast with on-board CT imaging [6]. Non-ablative RT has failed to improve overall survival (OS) in prospective randomized studies compared to chemotherapy alone and attempts to deliver ablative dose in five or fewer fractions with CTgRT have resulted in significant toxicity [5,7,8].

Stereotactic magnetic resonance image magnetic resonance-guided online adaptive radiation therapy (SMART) permits safe target dose escalation in five fractions through a superior soft tissue contrast of MRI, continuous intrafraction cine-MRI with automatic beam gating, and online adaptive replanning as demonstrated by the multi-center phase 2 SMART trial [9]. While online adaptive radiation therapy (oART) may facilitate safe dose escalation, there is a lack of data quantifying differences in dose-escalated outcomes among PDAC patients treated with and without oART. We, therefore, performed an in silico comparison of five-fraction ablative non-adapted CTgRT vs. SMART not only using the original simulation imaging, but also unique MRI scans acquired across each treatment fraction to characterize the impact of interfraction anatomic changes on target volume and GI OAR metrics.

## 2. Materials and Methods

### 2.1. Study Overview

After obtaining institutional review board approval, we evaluated the original simulation day and daily adapted SMART plans for 20 consecutive LAPC patients previously treated at our institution on a 0.35 Tesla (T) MRI-guided linear accelerator with five-fraction SMART. Each patient had MRI and CT simulation scans performed on the same day for treatment planning as per our institutional protocol [10]. These scans were performed back-to-back and temporally separated by 30–60 min in order to limit changes in internal anatomy. We retrospectively generated CTgRT plans using the original CT simulation scans as well as CTgRT plans using each treatment day MRI scan that was previously acquired for SMART delivery, which was able to clearly illustrate daily internal anatomic changes. CTgRT planning was performed using the same dose prescription and OAR constraints as the corresponding SMART plans. All CTgRT plans were created blinded to the results of the SMART plans with the assumption that CTgRT would be delivered on a standard linac without oART capability. CTgRT and SMART plans were then compared to assess the dosimetric impact of interfraction anatomic changes on target volumes and OARs.

### 2.2. SMART Simulation, Planning, and Treatment

All patients underwent conventional MRI simulation on a 0.35 T MR-Linac system (MRIdian, ViewRay Systems, Oakwood Village, OH, USA) using a previously described SMART institutional workflow [10]. MRI simulation was performed using true fast imaging with a steady-state free precession (TrueFISP) scan in inspiration breath hold (BH). While a 4D CT simulation (SOMATOM Definition Edge, Siemens Healthcare, Forchheim, Germany) was performed on the same day as MRI simulation, the 4D CT was not used for the patient’s SMART workflow. Segmentation was performed manually on the MRI simulation scan for baseline plans. For daily SMART plans, targets were rigidly propagated, and OARs were deformably propagated within the MRIdian system, followed by manual edits to the MRI of the day. The MRIdian’s deformation algorithm uses inverse consistent, free-form deformable image registration with a similarity metric correlation coefficient for the MRI simulation scan to the MRI of the day, respectively.

Treatment was administered using a step-and-shoot intensity modulated radiotherapy (IMRT) technique with continuous intrafraction cine-MRI, automatic beam gating, and oART as needed to meet OAR constraints. SMART plans typically were designed with ~18–28 fields and ~50–60 total segments [10,11]. All SMART plans were calculated with a 2 mm isotropic dose grid and Monte Carlo dose calculation algorithm with magnetic field corrections. Electron densities used for dose calculation included a bulk assignment for air (luminal gas), bone (vertebral body), and water (body) for the MR-only planning technique.

All patients were treated with a prescribed dose of 50/33 Gy to the gross tumor volume (GTV) and elective clinical target volume (CTV), respectively, using a simultaneous integrated boost technique. The CTV included at least a 5 mm isotropic margin around the GTV and at least the proximal 2–3 cm of the celiac axis and superior mesenteric artery. Isotropic 3 mm expansions of the GTV and CTV were performed to generate the 50 Gy and 33 Gy planning target volumes (PTVs), respectively. As all patients were treated with automatic beam gating, an internal target volume was not used.

The highest planning priority was to meet OAR constraints over target volume coverage. An isotoxicity approach was used in which plans were normalized to the nearest gastrointestinal (GI) OAR to optimize target volume coverage. The following OAR constraints were used to optimize the original simulation plan and each fractional adapted plan: stomach/duodenum/small bowel V35 ≤ 0.5 cc, V40 ≤ 0.03 cc; large bowel V38 ≤ 0.5 cc, V43 ≤ 0.03 cc; kidney mean < 10 Gy, liver mean < 13 Gy, and spinal cord V20 ≤ 0.03 cc [12]. For plan quality reporting, the initial plan dose and each fractional SMART plan dose was reported for target volumes and GI OARs.

### 2.3. CTgRT Planning Using Original Simulation Day Anatomy

Six CTgRT plans were retrospectively generated for each patient, one using the CT simulation scan acquired for SMART and 5 using the daily MRI scans acquired for SMART delivery that demonstrated interfraction anatomic changes. Thus, we evaluated a total of 120 CTgRT plans across the 20 patients. CTgRT planning was performed blinded to the results of the SMART plans.

Target volumes from MR simulation were rigidly propagated to the CT simulation scan. The average intensity projection CT constructed from the 4D CT scan was used as the primary scan for CTgRT segmentation and planning. Target volumes were reviewed and edited as necessary by a GI-specialized radiation oncologist to account for differences in GTV position based on CT-to-MRI image registration; these edits were typically very minor.

As expected, GI OAR anatomy was not identical on the MRI and CT simulation scans despite being acquired back-to-back on the same day, and, therefore, while the target volumes were based on the anatomy from the MR simulation scan, the GI OARs were contoured based on the anatomy from the CT simulation scan. Since treatment was assumed to be in BH, there was no internal target volume used for CTgRT plans just as none was used for SMART plans.

CTgRT planning used volumetric modulated arc therapy (VMAT) with identical prescription dose and OAR constraints from the SMART plans. The planning priorities for the CTgRT plans was the same as for the SMART plans, which was to meet OAR constraints and secondarily optimize target volume coverage. Treatment planning was performed in Eclipse Acuros version 15.6.06 (Varian Medical Systems, Palo Alto, CA, USA) on an isotropic 1.25 mm × 1.25 mm × 1.25 mm calculation grid. CTgRT plans used 2–3 full arcs (gantry angles 181–179 degrees) with collimator rotations of 30, 330, and 90 degrees using a high-definition multi-leaf collimator. The electron density for dose calculation were calculated through the conversion of HU to density from the nominal CT scan.

### 2.4. CTgRT Delivery Modeling Using Pre-Treatment MRI Imaging

Daily cone-beam CT (CBCT) scans were not acquired given that all patients were treated with SMART. We simulated the dose that would have been delivered with each fraction with CTgRT using the daily MRI scans acquired for SMART treatment. These MRI images provide superior soft tissue contrast compared to CBCT for the precise delineation of both gross tumor and GI OARs. To this end, the daily segmentation and MR image from each delivered SMART fraction was used to create the corresponding daily CTgRT plans. We assessed the predicted target volume and GI OAR doses across each of the 5 treatment days, assuming that the CTgRT plan created from the original simulation day anatomy was delivered without oART by rigidly registering the CT simulation scan to each treatment day MRI scan focusing on alignment to the GTV. The dose was rigidly propagated to the fractional MRI scan using Velcoity (Varian Medical Systems, Palo Alto, CA, USA), and dose statistics were reported for target volumes and GI OARs.

### 2.5. Statistical Analysis

Median values with standard deviations (SDs) were calculated for each target size (GTV, CTV, PTV50, and PTV33) along with dose evaluation parameters for each target (V100, D95, D90, D80, and mean dose). Median plus SD values for V35 Gy and V40 Gy were obtained for duodenum, small bowel, and stomach across all simulation and treatment plans. For the large bowel, V38 Gy and V43 Gy values were assessed. A forest plot was created for each OAR across fractions to compare CTgRT and SMART by plotting median values alongside their corresponding 95% confidence intervals. Overall median values without an initial plan for CT and MR across all fractions, target sizes, and organs were computed. Fractions that did not require oART were excluded. The Wilcoxon signed-rank test was utilized to identify statistically significant differences between the CTgRT and SMART for each target in terms of size and dose evaluation parameters across all fractions. *p*-values from the Wilcoxon test were utilized to evaluate the statistical significance of discrepancies between the CTgRT and SMART readings. A *p*-value below 0.05 was considered statistically significant. All statistical analyses were conducted utilizing SAS V9.4 (SAS Institute Inc., Cary, NC, USA).

## 3. Results

**Figure 1:**
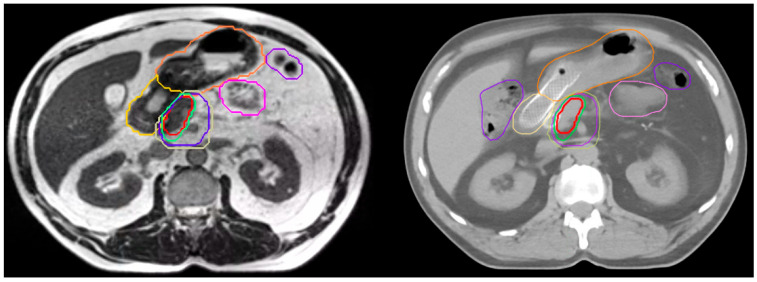
Sample patient contours on MRI (**left**) and CT (**right**) simulation scans: GTV (red), CTV (dark purple), PTV50 (green), PTV33 (tan), stomach (orange), duodenum (gold), small bowel (pink), large bowel (light purple).

A total of 240 plans (120 CTgRT, 120 SMART) were evaluated across the 20 patients with each patient having six CTgRT plans (one simulation plus five treatment days) and six SMART plans (one simulation plus five treatment days). Ninety percent of patients had tumors involving the pancreatic head. Median size (range) of SMART vs. CTgRT target volumes were GTV 27 cc (6–102) vs. 21 cc (4–98), CTV 97 cc (47–207) vs. 86 cc (42–190), PTV33 132 cc (37–268) vs. 144 cc (63–247), and PTV50 43 cc (18–141) vs. 40 cc (8–135). Figure 1 illustrates target and OAR contours for a sample patient. In all, 96% of treatment day plans with either CTgRT or SMART required oART to avoid a violation of one or more GI OAR constraints.

**Figure 2:**
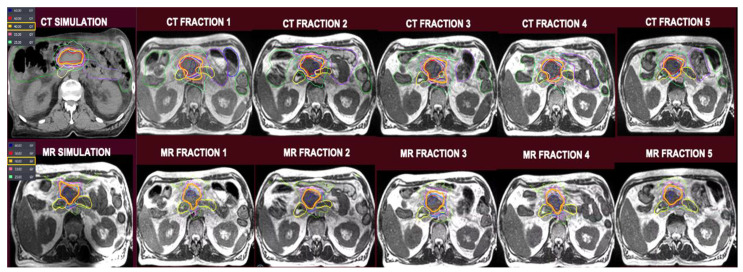
Top row, from left to right: CT simulation-based plan followed by CT plans superimposed on pre-treatment MRI scans. Bottom row, from left to right: MRI simulation-based plan followed by MRI plans superimposed on pre-treatment MRI scans.

Table 1 describes target volume coverages across all five treatment fractions between SMART vs. non-adapted CTgRT. There were no statistically significant differences for GTV, CTV, PT50, and PTV33 targets between the techniques. Despite re-planning to maintain GI OAR constraints, SMART plans maintained a V100% of 81.2% along with D95, D90, and D80 dose levels of 39.4 Gy, 45 Gy, and 50.4 Gy, respectively, for PTV50. Figure 2 shows isodose levels for CTgRT and SMART plans on simulation and pre-treatment MRI scans for a sample patient. 

**Figure 3:**
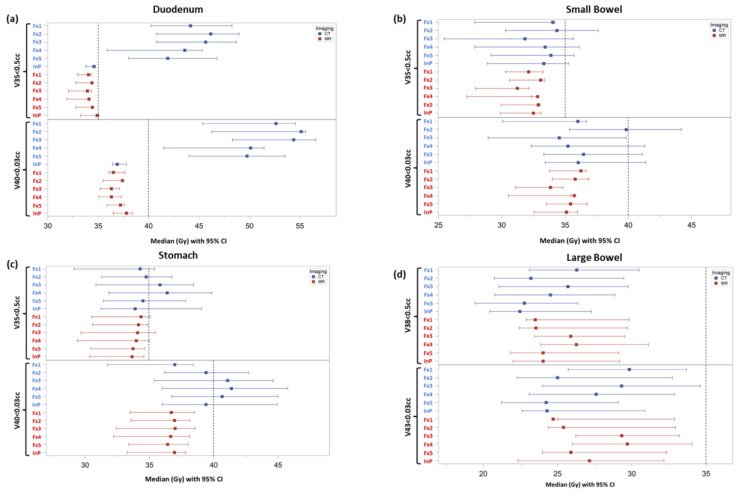
Forest plots of individual GI OARs displaying median dose levels with 95% confidence intervals for relevant dosimetric constraints achieved with initial simulation plans (InP) and treatment fraction plans (Fx1-5) using CTgRT (CT) vs. SMART (MR): (**a**) Duodenum V35 Gy and V40 Gy, (**b**) Small Bowel V35 and V40 Gy, (**c**) Stomach V35 Gy and V40 Gy, and (**d**) Large Bowel V38 Gy and V43 Gy. The horizonal dashed line represents the dose constraint for each respective GI OAR.

Figure 3 shows a forest plot for each luminal GI OAR comparing CTgRT and SMART doses at relevant dose constraints for initial and treatment day plans. Significant differences were observed in duodenum median V35 Gy ≤ 0.5 cc (34.2 vs. 41.9 Gy, *p* = 0.0035) and duodenum median V40 Gy ≤ 0.03 cc (37 vs. 52.5 Gy, *p* = 0.0006) constraints favoring SMART over CTgRT plans, respectively, across all treatment fractions. However, no significant median dose differences were observed between CTgRT and SMART plans for small bowel V35 Gy (31.7 vs. 33.2 Gy, *p* = 0.3) and V40 Gy (34.9 vs. 36.3, *p* = 0.18) or large bowel V38 Gy (24.1 vs. 24, *p* = 0.41) and V43 Gy (26.6 vs. 26, *p* = 0.56). Although median stomach V35 Gy differences were also non-significant (34.1 vs. 34.4, *p* = 0.28), the difference in stomach V4 Gy trended towards significance favoring SMART (37 vs. 40.3 Gy, *p* = 0.057).

## 4. Discussion

The variable proximity of GI OARs to target tissues remains a considerable obstacle for dose escalation in PDAC. Although MRI has long been known to provide a better soft tissue contrast and visualization of abdominal organs and tumors [13], CTgRT remains the predominant form of treatment for PDAC. To the best of our knowledge, this is the first study of ablative CTgRT and SMART in LAPC comparing target and OAR dose impacts from both approaches during treatments meeting criteria for adaptive re-planning. It is also the first study of its kind to use MRI to more accurately delineate target and OAR anatomy on each treatment day for both SMART and CTgRT plans.

Previously published data comparing CT vs. MRI based ablative SBRT for adrenal metastases demonstrated the need for adaptive therapy in 61% vs. 45% of patients, respectively. The lower adaptive rate with CT planning was presumably due to the steeper and more conformal dose gradient afforded by VMAT vs. step-and-shoot IMRT used in MRI planned therapy [14]. Our study in LAPC showed high rates of GI OAR violations (96%) for both CT and MRI plans without oART, which may be explained by the high percentage of pancreatic head tumors (90%) in close proximity to the duodenum and stomach. This remains consistent with data from the phase 2 SMART trial in which 93.1% of treated fractions required oART to achieve ablative dose levels for 136 LAPC cases [9]. Similarly, the need for oART was also observed in one of the first reported series of pancreatic patients treated with dose escalated SMART by Hassandzadeh et al., in which 93% of 220 treated fractions required adaptation to permit safe dose escalation [15]. The most common indication for adaptive therapy was excess dose to the duodenum and small bowel, which was observed in 68% and 38% of fractions, respectively. In addition to the previously stated high percentage of head tumors in our cohort, the paucity of small bowel violations observed in our subjects may also be attributed to the larger 5 mm PTV margin employed by the group from Washington University in comparison to the 3 mm PTV used in the current study.

Researchers from M.D. Anderson Cancer Center recently published a retrospective analysis of CTgRT SBRT plans for 18 borderline resectable and locally advanced PDAC patients, initially treated to doses of 33 to 40 Gy in five fractions but replanned to ablative dose levels [16]. Target structures included both the primary pancreatic tumor and tumor vessel interface, treated collectively to a dose of 50 Gy. Although the D95% GTV dose remained above 48 Gy with replanning, they noted that OAR constraints were violated in the majority of cases when escalating to ablative thresholds without adapting for interfraction anatomic changes. Using fan-beam CT-on-rails, which provided improved image quality compared to cone beam CT (CBCT), they contended that adaptive therapy could be performed with CT guidance while maintaining OAR constraints. This analysis provides a potential framework for dose escalation and adaptive therapy using a CT-only approach but requires clinical validation.

Published attempts at dose escalation in five or fewer fractions with CT guidance, in the absence of adaptive therapy, have demonstrated high rates of treatment-related toxicities [8,17]. Hoyer et al. evaluated 22 LAPC patients treated to 45 Gy in three fractions (BED_10_ = 112.5 Gy) on a phase 1–2 protocol. Median survival time was only 5.7 months with almost a quarter of the patients experiencing severe toxicities including stomach/duodenal ulceration or perforation within 2 weeks of treatment [17]. Courtney et al. completed a phase one-dose escalation study with 40, 45, or 50 Gy CT-guided SBRT in five fractions for 30 patients with unresectable or metastatic pancreatic cancer [8]. They noted “nontrivial rates of late gastrointestinal toxicity,” including a 7% risk grade 4 or 5 events with doses of 45 Gy or more.

More recent, emerging data suggests that dose escalation to 40 Gy in five fractions (BED10 = 72 Gy) may be achieved in LAPC by combining CTgRT with oART [18]. Despite limiting dose to 25 Gy where PTV crossed within 5 mm of a GI OAR, 98% of patients in this series required oART to meet constraints. A median overall survival of 21.6 months was reported, but with a 19% (4/21) rate of late grade ≥ 3 toxicity. Prior to this report, CT-guided ablative therapy for LAPC with acceptable toxicity rates had only been achieved using conventional or hypofractionated techniques. Krishnan et al. reported a 36% vs. 19% overall survival at 2 years without any added toxicity for patients treated to a BED10 > 70 vs. ≤70 [19] These patients were treated using a variety of fractionation schemes with the most common being 63–70 Gy in 28 fractions, 67.5 Gy in 15 fractions, and 51.3–70.4 Gy in 13–39 fractions. Reyngold et al. shared a cohort study of 119 patients treated to 67.5 Gy in 15 fractions, or 75 Gy in 25 fractions if tumors were located within 1 cm of GI luminal structures [20]. Pre-treatment CBCT imaging and respiratory management were utilized to improve treatment accuracy. Two-year overall survival was 38% with a 12.6% risk of grade 3 GI toxicity. In contrast, the SMART trial used 50 Gy in five-fraction ablative therapy with daily pre-treatment MRI imaging and intrafraction monitoring to permit adaptive therapy. This approach yielded a 54% two-year survival with late grade ≥3 GI toxicity below 5% [9,21].

Our analysis is limited by several factors. Firstly, there were small differences in CTgRT vs. SMART target volumes since they were manually edited to account for soft tissue differences that resulted from the rigid registration of the CT and MRI scans. There also are known differences in target volume delineation for pancreatic cancer based on CT versus MRI. Although MRI and CT scans used for contouring were completed the same day, slight variations in the relative positioning of OARs and targets were present as expected. Additionally, CTgRT targets were contoured retrospectively using average intensity projection CTs constructed from 4 D CT scans. In contrast, SMART contours were completed using MRI scans completed during breath hold. Nonetheless, median CTgRT volumes were smaller for GTV, CTV, and PTV50 volumes compared to their SMART counterparts, potentially leading to lower OAR doses from smaller isodose clouds. Therefore, the benefits from SMART may actually be underestimated. On the other hand, abdominal compression, which is routinely used in CT-based SBRT for LAPC, was not employed in our patients. This may permit increased variance in OAR positioning relative to target volumes with an overestimation of interfraction anatomic changes. Lastly, we did not account for added on-table time required for oART during SMART. The extra treatment time, though mitigated by intrafraction tracking, may provide a greater opportunity for anatomical changes, which may limit advantages observed with SMART.

## 5. Conclusions

This is the first evaluation of interfraction anatomic changes on predicted GI OAR doses for ablative, five-fraction CTgRT vs. SMART for PDAC. Both approaches yielded comparable target coverage. However, GI OAR constraint violations were observed in 96% of treatment plans using CT or MRI planning, highlighting the importance of adaptive re-planning. Although oART use is routine on MRI-guided linacs, it is not commonly available on CT-guided units. Our analysis uniquely employed MRI imaging to compare treatment day anatomy and predicted dose with CTgRT vs. SMART for LAPC. We demonstrated significant GI OAR-predicted dose violations without the use of oART, which is in line with significant GI toxicity demonstrated from prior dose-escalated SBRT trials that prescribed an ablative dose. Early data of CTgRT with oART are promising, but further studies with longer follow up are needed given the reported rate of late toxicities. In summary, five-fraction ablative SBRT for LAPC should be limited to centers that are able to treat with oART. If oART is not available, then dose escalation should instead be delivered using moderate hypofractionation (e.g., 75 Gy in 25 fractions or 67.5 Gy in 15 fractions). These ablative approaches should be prioritized in patients with SMAD4 expressing tumors demonstrating an increased risk of local failure after conventional radiotherapy [4].

## Figures and Tables

**Table 1:** Median target volume dose parameters for treatment fractions 1–5 using CTgRT vs. SMART.

	Target	CTgRT	SMART	*p*-Value
V100%	GTV	92.4	92.2	0.8178
	CTV	98.4	99.1	0.1555
	PTV50	79.9	81.2	0.5075
	PTV33	94.1	95.5	0.5792
D95 (Gy)	GTV	48.0	47.0	1.0000
	CTV	34.8	34.9	0.7557
	PTV50	40.2	39.4	0.7352
	PTV33	32.6	33.1	0.4016
D90 (Gy)	GTV	51.3	52	0.6359
	CTV	36.3	35.9	0.7972
	PTV50	44.0	45.0	0.8817
	PTV33	34.0	34.1	1.0000
D80 (Gy)	GTV	54.0	55.2	0.5074
	CTV	38.1	38.1	0.9784
	PTV50	50.2	50.4	0.4407
	PTV33	36.0	36.0	0.8604
Mean Dose (Gy)	GTV	57.7	59.0	0.3104
	CTV	47.8	48.2	0.9246
	PTV50	54.9	56.1	0.2792
	PTV33	45.2	45.1	0.9031

## Data Availability

Research data are stored in an institutional repository and will be made available upon request to the corresponding authors.

## Abbreviations

The following abbreviations are used in this manuscript:

CTgRT	Computed Tomography-Guided Radiotherapy
CTV	Clinical Target Volume
GTV	Gross Tumor Volume
LAPC	Locally Advanced Pancreatic Cancer
oART	Online Adaptive Radiation Therapy
PDAC	Pancreatic Ductal Adenocarcinoma
PTV	Planning Target Volume
SBRT	Stereotactic Body Radiation Therapy
SMART	Stereotactic Magnetic Resonance-Guided Online Adaptive Radiation Therapy

## References

[1] Siegel R.L., Kratzer T.B., Giaquinto A.N., Sung H., Jemal A. (2025). Cancer statistics, 2025. CA Cancer J. Clin..

[2] Hu J.-X., Zhao C.-F., Chen W.-B., Liu Q.-C., Li Q.-W., Lin Y.-Y., Gao F. (2021). Pancreatic cancer: A review of epidemiology, trend, and risk factors. World J. Gastroenterol..

[3] Iacobuzio-Donahue C.A., Fu B., Yachida S., Luo M., Abe H., Henderson C.M., Vilardell F., Wang Z., Keller J.W., Banerjee P. (2009). DPC4 gene status of the primary carcinoma correlates with patterns of failure in patients with pancreatic cancer. J. Clin. Oncol..

[4] Crane C.H., Varadhachary G.R., Yordy J.S., Staerkel G.A., Javle M.M., Safran H., Haque W., Hobbs B.D., Krishnan S., Fleming J.B. (2011). Phase II trial of cetuximab, gemcitabine, and oxaliplatin followed by chemoradiation with cetuximab for locally advanced (T4) pancreatic adenocarcinoma: Correlation of Smad4 (Dpc4) immunostaining with pattern of disease progression. J. Clin. Oncol..

[5] Hammel P., Huguet F., van Laethem J.-L., Goldstein D., Glimelius B., Artru P., Borbath I., Bouché O., Shannon J., André T. (2016). Effect of chemoradiotherapy vs chemotherapy on survival in patients with locally advanced pancreatic cancer controlled after 4 months of gemcitabine with or without erlotinib: The LAP07 randomized clinical trial. JAMA.

[6] Lee J., Dean C., Patel R., Webster G., Eaton D.J. (2019). Multi-center evaluation of dose conformity in stereotactic body radiotherapy. Phys. Imaging Radiat. Oncol..

[7] Fietkau R., Ghadimi M., Grützmann R., Wittel U.A., Jacobasch L., Uhl W., Croner R.S., Bechstein W.O., Neumann U.P., Waldschmidt D. (2022). Randomized phase III trial of induction chemotherapy followed by chemoradiotherapy or chemotherapy alone for nonresectable locally advanced pancreatic cancer: First results of the CONKO-007 trial. Am. Soc. Clin. Oncol..

[8] Courtney P.T., Paravati A.J., Atwood T.F., Raja N., Zimmerman C.T., Fanta P.T., Lowy A.M., Simpson D.R., Xu R., Murphy J.D. (2021). Phase I trial of stereotactic body radiation therapy dose escalation in pancreatic cancer. Int. J. Radiat. Oncol. Biol. Phys..

[9] Chuong M.D., Lee P., Low D.A., Kim J., Mittauer K.E., Bassetti M.F., Glide-Hurst C.K., Raldow A.C., Yang Y., Portelance L. (2024). Stereotactic MR-guided on-table adaptive radiation therapy (SMART) for borderline resectable and locally advanced pancreatic cancer: A multi-center, open-label phase 2 study. Radiother. Oncol..

[10] Mittauer K.E., Yarlagadda S., Bryant J.M., Bassiri N., Romaguera T., Gomez A.G., Herrera R., Kotecha R., Mehta M.P., Gutierrez A.N. (2023). Online adaptive radiotherapy: Assessment of planning technique and its impact on longitudinal plan quality robustness in pancreatic cancer. Radiother. Oncol..

[11] Mittauer K.E., Hill P.M., Bassetti M.F., Bayouth J.E. (2020). Validation of an MR-guided online adaptive radiotherapy (MRgoART) program: Deformation accuracy in a heterogeneous, deformable, anthropomorphic phantom. Radiother. Oncol..

[12] Chuong M.D., Bryant J., Mittauer K.E., Hall M., Kotecha R., Alvarez D., Romaguera T., Rubens M., Adamson S., Godley A. (2021). Ablative 5-fraction stereotactic magnetic resonance–guided radiation therapy with on-table adaptive replanning and elective nodal irradiation for inoperable pancreas cancer. Pract. Radiat. Oncol..

[13] Khoo V., Joon D. (2006). New developments in MRI for target volume delineation in radiotherapy. Br. J. Radiol..

[14] Rodriguez L.L., Kotecha R., Tom M.C., Chuong M.D., Contreras J.A., Romaguera T., Alvarez D., McCulloch J., Herrera R., Hernandez R.J. (2022). CT-guided versus MR-guided radiotherapy: Impact on gastrointestinal sparing in adrenal stereotactic body radiotherapy. Radiother. Oncol..

[15] Hassanzadeh C., Rudra S., Bommireddy A., Hawkins W.G., Wang-Gillam A., Fields R.C., Cai B., Park J., Green O., Roach M. (2021). Ablative five-fraction stereotactic body radiation therapy for inoperable pancreatic cancer using online MR-guided adaptation. Adv. Radiat. Oncol..

[16] Rhee D.J., Beddar S., Abi Jaoude J., Sawakuchi G., Martin R., Perles L., Yu C., He Y., Court L.E., Ludmir E.B. (2023). Dose escalation for pancreas SBRT: Potential and limitations of using daily online adaptive radiation therapy and an iterative isotoxicity automated planning approach. Adv. Radiat. Oncol..

[17] Hoyer M., Roed H., Sengelov L., Traberg A., Ohlhuis L., Pedersen J., Nellemann H., Berthelsen A.K., Eberholst F., Engelholm S.A. (2005). Phase-II study on stereotactic radiotherapy of locally advanced pancreatic carcinoma. Radiother. Oncol..

[18] Lee A., Pasetsky J., Lavrova E., Wang Y.-F., Sedor G., Li F.L., Gallitto M., Garrett M., Elliston C., Price M. (2024). CT-guided online adaptive stereotactic body radiotherapy for pancreas ductal adenocarcinoma: Dosimetric and initial clinical experience. Clin. Transl. Radiat. Oncol..

[19] Krishnan S., Chadha A.S., Suh Y., Chen H.-C., Rao A., Das P., Minsky B.D., Mahmood U., Delclos M.E., Sawakuchi G.O. (2016). Focal radiation therapy dose escalation improves overall survival in locally advanced pancreatic cancer patients receiving induction chemotherapy and consolidative chemoradiation. Int. J. Radiat. Oncol. Biol. Phys..

[20] Reyngold M., O’Reilly E.M., Varghese A.M., Fiasconaro M., Zinovoy M., Romesser P.B., Wu A., Hajj C., Cuaron J.J., Tuli R. (2021). Association of ablative radiation therapy with survival among patients with inoperable pancreatic cancer. JAMA Oncol..

[21] Parikh P.J., Lee P., Low D.A., Kim J., Mittauer K.E., Bassetti M.F., Glide-Hurst C.K., Raldow A.C., Yang Y., Portelance L. (2023). A multi-institutional phase 2 trial of ablative 5-fraction stereotactic magnetic resonance-guided on-table adaptive radiation therapy for borderline resectable and locally advanced pancreatic cancer. Int. J. Radiat. Oncol. Biol. Phys..

